# SSAO/VAP-1 in Cerebrovascular Disorders: A Potential Therapeutic Target for Stroke and Alzheimer’s Disease

**DOI:** 10.3390/ijms22073365

**Published:** 2021-03-25

**Authors:** Mercedes Unzeta, Mar Hernàndez-Guillamon, Ping Sun, Montse Solé

**Affiliations:** 1 Department of Biochemistry and Molecular Biology, Institute of Neurosciences, Universitat Auònoma de Barcelona, 08193 Barcelona, Spain; Mercedes.Unzeta@uab.es; 2 Neurovascular Research Laboratory, Vall d’Hebron Research Institute, Universitat Autònoma de Barcelona, 08035 Barcelona, Spain; spmontse2@gmail.com; 3 Department of Neurology, Pittsburgh Institute of Brain Disorders and Recovery, University of Pittsburgh School of Medicine, Pittsburgh, PA 15213, USA; sunp@upmc.edu

**Keywords:** SSAO/VAP-1, stroke, Alzheimer’s disease, vascular damage, blood–brain barrier dysfunction, neurovascular unit, inflammation, oxidative stress

## Abstract

The semicarbazide-sensitive amine oxidase (SSAO), also known as vascular adhesion protein-1 (VAP-1) or primary amine oxidase (PrAO), is a deaminating enzyme highly expressed in vessels that generates harmful products as a result of its enzymatic activity. As a multifunctional enzyme, it is also involved in inflammation through its ability to bind and promote the transmigration of circulating leukocytes into inflamed tissues. Inflammation is present in different systemic and cerebral diseases, including stroke and Alzheimer’s disease (AD). These pathologies show important affectations on cerebral vessels, together with increased SSAO levels. This review summarizes the main roles of SSAO/VAP-1 in human physiology and pathophysiology and discusses the mechanisms by which it can affect the onset and progression of both stroke and AD. As there is an evident interrelationship between stroke and AD, basically through the vascular system dysfunction, the possibility that SSAO/VAP-1 could be involved in the transition between these two pathologies is suggested. Hence, its inhibition is proposed to be an interesting therapeutical approach to the brain damage induced in these both cerebral pathologies.

## 1. Introduction

### 1.1. SSAO: An Amine Oxidase

Amine oxidases (AOs) are a group of enzymes that catalyze the oxidative deamination of various amines from the endogenous and xenobiotic origin, as well as those present in the diet. According to their attached cofactors, the amine oxidases include the following categories: (i) flavin adenine dinucleotide (FAD)-containing enzymes (E.C. 1.4.3.4) include polyamine oxidase (PAO) (E.C. 1.5.3.11) [1], and both isoforms of Monoamine oxidase (MAO-A and -B), which participate in the metabolism of biogenic amines with neurotransmitter functions [2,3]; and (ii) topa-quinone (TPQ) or lysine tyrosyl quinone (LTQ)-containing enzymes, which makes them easily inhibited by carbonyl compounds, such as semicarbazide, including diamine oxidase (DAO) (E.C. 1.4.3.22), lysyl oxidase (LOX) (E.C. 1.4.3.13), and soluble and membrane-bound semicarbazide sensitive amine oxidase (SSAO) (E.C. 1.4.3.21) [4,5,6].

All AOs are able to catalyze the oxidative deamination of different amines, according to the following reaction:

R-CH_2_-NH_2_ + O_2_ + H_2_O → R-CHO + NH_3_ + H_2_O_2_

SSAO is multifunctional. It can metabolize the deamination of primary amines exclusively, and its functions depend on the tissue that expresses it [7], as listed in Table 1. Some SSAO substrates, such as benzylamine, show an overlap with MAO substrates, responsible for the primary, secondary, and tertiary amine metabolism, but the physiological substrates methylamine and aminoacetone are exclusively metabolized by SSAO [8,9,10]. Physiologically, methylamine can be derived from the metabolism of epinephrine [11], adrenaline [12], creatine, creatinine [13,14], sarcosine and choline [15,16,17]. Methylamine may also come from the digestion of food and beverages or cigarette smoking [18]. Aminoacetone, however, is a metabolic product of glycine and threonine [19]. SSAO can catalyze the deamination of methylamine and aminoacetone to generate not only hydrogen peroxide (H_2_O_2_) and ammonia but also formaldehyde and methylglyoxal, respectively.

When overproduced, the metabolites generated by the deamination of SSAO substrates may constitute potentially hazardous products; thus, the inhibition of SSAO activity may be beneficial under some pathological conditions, which will be detailed further below. Many chemicals can inhibit both SSAO and MAO activities [20]. For example, the hydrazine derivatives phenelzine, phenylhydrazine, hydralazine, benserazide, and carbidopa, can inhibit SSAO activity, and some of them also inhibit MAO activity [20,21]. Lately, the hypothesis that SSAO inhibition could become a therapeutic target in several diseases has significantly increased the design and synthesis of new specific molecules that are able to modulate or inhibit its activity [22,23,24,25,26].

Molecular modeling studies showed that the membrane-bound SSAO in humans is a 180 kDa homodimeric glycoprotein composed of two identical 90 kDa monomer subunits. It consists of a short membrane spinning domain and three catalytically active extracellular copper-containing amine oxidase domains [27,28]. Surprisingly, the cloning of a novel cell adhesion molecule, endothelial vascular adhesion protein-1 (VAP-1) cDNA, shows that VAP-1 not only has SSAO activity but is also significantly identical to copper-containing amine oxidase [29]. Since then, VAP-1 has been considered to have dual functions and is defined as a new type of adhesion molecule with both cell adhesion function and SSAO enzyme activity, as SSAO/VAP-1 [30].

**Table 1:** Semicarbazide-sensitive amine oxidase (SSAO)/ vascular adhesion protein-1 (VAP-1) tissue localization, cell type expression, physiological substrates proved to be metabolized by these tissues and physiological function. Note the absence of SSAO/VAP-1 expression in cerebral parenchymal cells (neurons and glia). Benzylamine is a non-physiological substrate of SSAO metabolized by SSAOs from different origins. Data are summarized from [7,8,9,10,11,12,13,14,15,16,17,18,19,31,32,33,34,35,36,37,38,39,40,41,42,43,44].

Tissue	Cell Type	Substrate	Function
Cerebrovascular tissue (meninges and microvessels) (human, rabbit, mouse, bovine)	Endothelial cells Smooth muscle cells	Methylamine (derived from epinephrine, adrenaline, creatine, sarcosine and choline) aminoacetone (derived from glycine and threonine)	Scavenger of endogenous dietary amines Generation of H_2_O_2_ as a signaling molecule Leukocyte trafficking under inflammation
Vascularized tissues (heart, kidney, lung, intestine, liver, retina and lymph nodes) and blood vessels (human, pig, rat, rabbit, bovine)	Endothelial cells Smooth muscle cells Pericytes	Phenylethylamine Dopamine Methylamine Tyramine Tryptamine	Metabolism of physiological circulating amines and xenobiotic ones Leukocyte binding and extravasation under inflammatory conditions
Adipose tissue (human and rat)	Adipocytes (white and brown)	Various endogenous and exogenous amines	Metabolism of endogenous amines Insulinomimetic effects through the generation of H_2_O_2_
Ureter and vas deferens	Non-vascular smooth muscle cells	Dopamine	Metabolism of physiological amines and xenobiotic ones
Endometrium (human)	Pericytes	Methylamine	Recruiting innate immune cells
Skin (guinea pig)	Fibroblasts	Histamine 1–4 Methylhistamine	Metabolism of physiological amines and xenobiotic ones
Dental pulp (human, pig)	Odontoblasts	Serotonin Phenylethylamine Tyramine Tryptamine	Contribution to inflammatory response in dental pulp (pulpitis)

### 1.2. SSAO/VAP-1: Expression and Tissular Localization

SSAO/VAP-1 is present in a wide number of mammalian species [31], both as a membrane-bound form and also as a soluble form in blood plasma [7]. High SSAO activity is observed to be associated with vascularized tissues [32], and specifically in blood vessels [33], where it is expressed by smooth muscle cells and endothelial cells [34].

Human SSAO/VAP-1, as in other species, is widely distributed in almost all vascularized tissues and adipose tissue. In humans, the presence of the SSAO enzyme also has been specifically reported in vascular endothelial cells and smooth muscle cells extracted from skin, heart, liver, kidney, and brain [35,36]. Its presence has also been described in pig dental pulp [37], where it is able to metabolize serotonin [38,39]. Regarding the cerebrovascular tissue, its presence in human and bovine brain meninges and microvessels, as well as in retina and eye sclera, were confirmed by using a rabbit anti-bovine lung SSAO/VAP-1 polyclonal antibody [40,41,45,46,47].

At the subcellular level, SSAO/VAP-1 located at the plasmatic cell membrane appears randomly distributed in structures named caveolae [48,49], constituting the lipid rafts. Whether it has a specific function related to this localization needs to be addressed, as lipid rafts are specialized membrane microdomains with a role in signal transduction that assemble signaling molecules and influence membrane protein trafficking [50].

### 1.3. SSAO/VAP-1 Physiological Functions

Up to now, several functions of SSAO/VAP-1 have been described under physiological conditions, as summarized in Table 1, related to its ability to metabolize primary amines: the protection against endogenous/xenobiotic amines, the local generation of signaling molecules, glucose transportation, and leukocyte trafficking under inflammation [8,51].

#### 1.3.1. Amine Deamination

The first main function attributed to SSAO/VAP-1 was based on its enzymatic activity, and it was associated with the homeostatic removal or scavenging of physiological active endogenous and xenobiotic amines, which are potentially hazardous [52]. SSAO deamination of its substrates can reduce the biological activities of the substrates. Moreover, all the catalytic products of SSAO have biological activities and may have important roles at physiological concentrations, contributing to the other functions attributed to SSAO/VAP-1. In this regard, low concentrations of H_2_O_2_ can behave as intracellular second messengers to participate in ligand stimulation, cell growth or cell death regulation, or NF-κB signaling activation. Subsequently, NF-κB activation modulates the expression of many other genes, such as MMPs, cytokines, chemokines, and vascular cell adhesion molecules [5,53]. This signaling is also involved in angiogenesis and cellular differentiation. It has also been demonstrated that H_2_O_2_ can activate mitogen-activated protein kinase (MAPK) as well as the c-Jun amino-terminal kinase (JNK) [54]. Furthermore, immunohistochemical studies indicate that in most human peripheral tissues, SSAO/VAP-1 might participate in the regulation of physiological processes through H_2_O_2_ generation [36].

#### 1.3.2. Activation of Glucose Transport

In isolated rat cardiac myocytes and rat adipocytes endosomal vesicles, insulin can induce the recruitment of the intracellular glucose transporters (GLUT4) and (GLUT1) [55]. Vesicle immunoisolation analysis indicated that GLUT4-containing vesicles from rat adipocytes contain substantial levels of SSAO activity and immunoreactive SSAO/VAP-1 protein. Furthermore, it has been reported that the SSAO substrate benzylamine could accelerate the transportation of glucose in the presence of low concentrations of vanadate in isolated adipocytes from rats. This effect of benzylamine and vanadate on glucose transport was totally abolished in the presence of semicarbazide, used as an SSAO inhibitor [56,57]. Later on, it was reported that some SSAO substrates stimulate glucose transport and inhibit lipolysis in human adipocytes, which confirmed the insulin-mimetic action of this protein, depending on its enzymatic activity [42].

#### 1.3.3. Leukocyte Adhesion Function under Inflammation

SSAO/VAP-1 can act as an adhesion protein in the leukocyte trafficking process [43,58,59,60,61]. Firstly, VAP-1 was reported to be involved in the human lymphocyte trafficking to high endothelial venules (HEVs) in the tonsils, peripheral lymph nodes (PLN), and inflamed synovia [29]. Besides, SSAO/VAP-1 was also found to have increased expression at the inflammation sites to control the recirculation of lymphocytes and the entry of leukocytes [43]. By establishing the primary endothelial cells with the expression of enzymatically active SSAO/VAP-1, firm evidence demonstrated that the SSAO activity of VAP-1 was directly involved in the rolling and transmigration steps during leukocyte adhesion [5,44]. SSAO/VAP-1 participates in these processes by acting as an adhesion protein but also through the SSAO-catalyzed end products [62]. In this regard, the H_2_O_2_ generated by its enzymatic activity is able to induce the expression of other endothelial adhesion molecules, such as MadCAM-1, E-selectin, P-selectin and CXCL8, through the activation of NF-kB [63]. This dual function of SSAO/VAP-1 also results in a different selectivity in the type of leukocytes to bind, which also depends on the organ and the type of inflammatory stimulus, as detailed in Table 2. It has been reported that lipopolysaccharide (LPS), interleukin 1β (IL-1β), interferon-γ (IFN-γ) and tumor necrosis factor-α (TNF-α) are the inflammatory mediators responsible of the SSAO/VAP-1 migration from intracellular vesicles to the plasma membrane [64]. Once there, different adhesion molecules facilitate leukocytes binding to SSAO/VAP-1 for a successful emigration cascade, such as peripheral lymph node addressin (PNAd), vascular cell adhesion molecule 1 (VCAM-1), or intracellular adhesion molecules (ICAM-1, ICAM-2) [43].

### 1.4. SSAO/VAP-1 Involvement in Pathological Conditions

The physiological functions of SSAO/VAP-1 can lead to a harmful situation when its levels are increased, as summarized in Table 3. Due to the potentially hazardous activity of the SSAO activity products, increased activity of this enzyme is associated with diverse human pathological processes. The SSAO metabolic products, such as formaldehyde or methylglyoxal, are toxic at high concentrations, especially in blood vessels [18,85]. In this regard, the in vitro treatment of vascular cells with methylamine, which generates formaldehyde, induces a dose- and time-dependent cytotoxic effect and activates apoptotic cell death through the tumor suppressor protein p53 activation, inducing PUMA-alpha expression, altering the mitochondrial Bcl-2 family proteins, and activating final effector caspases [86]. In the case of the substrate aminoacetone, the generation of methylglyoxal by SSAO activity has been implicated in vascular alterations, and it is a well-known precursor of advanced glycation end products (AGEs), which are involved in diabetic complications and vascular degeneration [87,88].

In humans and other species, soluble SSAO/VAP-1 exists in the serum of healthy adults [89,90], but its levels are found to increase in several pathological conditions. Experiments performed in adipocytes evidenced that soluble SSAO/VAP-1 could be shed from the membrane-bound form depending on a matrix metalloproteinase (MMP) activity in diabetic and obese animals [105]. It seems that under pathological conditions, soluble SSAO/VAP-1 originates from adipocytes, endothelial cells and smooth muscle cells [106], but given that various cellular sources can secrete different types of MMPs, such as neurons, cerebral microvascular endothelial cells, astrocytes, and inflamed neutrophils, which kinds of MMPs participate in the shedding of soluble SSAO/VAP-1 still warrant future investigation.

Plasma soluble SSAO/VAP-1 is increased in various systemic diseases: in diabetes, atherosclerosis [107,108,109,110], congestive heart failure [91] and non-diabetic morbidity obesity [111]. Moreover, it has also been described that the soluble SSAO/VAP-1 is increased in malignant hypertension [51], inflammatory diseases (cirrhotic liver inflammation) [89], and retinopathies associated with diabetes mellitus [112]. The specific mechanisms regulating the soluble plasmatic SSAO/VAP-1 and activity increase in these pathologic conditions are still not fully elucidated. Since soluble SSAO/VAP-1 may be derived from the membrane-bound form, the enhanced SSAO activity in plasma may be attributed to upregulated expression of membrane-bound SSAO/VAP-1 in diabetic patients [17] in response to inflammation [89]. The increasing prevalence of chronic inflammatory and autoimmune diseases associated with the aging population points out the interest in developing therapies directed against SSAO/VAP-1 for the treatment of chronic inflammatory diseases [113].

On the other hand, plasma SSAO activity was found to decrease in severely burnt or cancer patients [114]. Using an experimental model of breast cancer in rats induced by 7,12-dimethylbenz(alpha)anthracene (DMBA), a decreasing SSAO activity was observed and correlated with cancer malignancy [115]. However, it was described that high levels of SSAO/VAP-1 are closely linked to alternative M2 macrophage activation during human glioma progression [116]. Moreover, the SSAO/VAP-1 expression in different astrocytoma grades and its correlation with clinicopathological features as well as prognosis of astrocytoma patients was studied. The expression of this enzyme was assayed by immunohistochemistry, and the level of SSAO/VAP-1 was found significantly higher in diffuse astrocytoma than those of pilocytic astrocytoma. Therefore, the authors concluded that SSAO/VAP-1 could be a promising prognostic biomarker in human astrocytoma [117].

SSAO/VAP-1 also has been found altered in central nervous system (CNS) pathologies. In humans, the concentration of soluble SSAO/VAP-1 in serum is significantly higher in multiple sclerosis (MS) patients with ongoing inflammatory activity, as demonstrated by gadolinium-enhancing MRI lesions [118]. Results suggest that SSAO/VAP-1 may participate in controlling leukocyte entry into the inflamed brain in this pathologic condition. In addition, the expression of membrane-bound SSAO/VAP-1 has been studied in focal rat models of experimental autoimmune encephalomyelitis (EAE) mimicking MS. Results reveal that SSAO/VAP-1 is expressed and is functionally active in vasculature within the induced focal EAE lesions during the acute phase of inflammation, and it remains expressed after the acute inflammation has subsided. These data support that SSAO/VAP-1 is actively involved in the development of inflammatory CNS lesions [119], thus becoming an interesting target to study its involvement in human vascular and inflammatory pathologies [120]. The involvement of SSAO/VAP-1 in stroke and AD, where its levels are found also elevated, will be discussed in-depth in the following sections.

## 2. Cerebrovascular Dysfunction in Stroke and AD

### 2.1. The BBB and Cerebrovascular Dysfunction

Brain endothelial cells are unique, as they are interconnected by focal adhesions known as “tight junctions”, resulting in a highly selective barrier, the blood-brain barrier (BBB). This constitutes a highly specialized endothelial membrane lining cerebral microvessels with the astrocyte end-feet and pericytes [121]. BBB effectively regulates the passive exchange of solutes, transporter-mediated substances (e.g., glucose, amino acids, ions), signaling molecules, and the trafficking of macromolecules (e.g., proteins, peptides) between the blood and the brain [122]. It also regulates the entry of leukocytes and plasma components into the brain and ensures the exclusion of neurotoxic molecules [123,124,125]. The brain takes about 20% of the overall glucose and oxygen of the body, and brain microvessels are responsible for the delivery of both substrates to brain parenchyma. Therefore, a link between cerebrovascular alterations and neurodegeneration seems plausible [126,127]. Vast bibliography points out the interrelationship between cerebrovascular tissue and neurodegeneration, as well as confirms the age-dependent deterioration of the BBB during normal aging in the hippocampus, the brain region that is responsible for learning and memory, but faster degradation in patients with mild cognitive impairment (MCI) compared with neurologically intact controls [128,129,130,131].

At the functional level, the BBB is integrated into the neurovascular unit (NVU), composed of glial cells (astrocytes and microglia) and brain vascular cells (pericytes, endothelial cells, and vascular smooth muscle cells). NVU acts as a complex tissue with all its cells communicating with each other by secreting molecular factors named angioneurins. This communication allows the regulation of BBB integrity, angiogenesis, neuroprotection, vascular perfusion, and synaptic plasticity, thus ensuring the correct development, maintenance, and function of the unit in a healthy brain [132,133,134,135]. On the other hand, the BBB/NVU dysfunction induces inadequate nutrient supply, accumulation of toxins in the brain, or altered secretion of proteins by NVU cells, which induce inflammation, oxidative stress, and neuronal damage. Dysfunction of the NVU is related to several CNS pathologies, such as ischemic and hemorrhagic stroke, tauopathies, MS, diabetic retinopathy, and HIV-1 infection [136,137,138].

Cerebrovascular inflammation is underlying during the progression of different CNS disorders, including AD, stroke, traumatic brain injury, etc. Under normal physiological conditions, the BBB prevents adhesion molecules trafficking into the brain, however under pathophysiological conditions, the BBB integrity is impaired and different adhesion molecules, such as chemokines, selectins, and vascular cell adhesion molecules (CAMs), enter the CNS and pathologically enhance neuroinflammation [139,140].

### 2.2. Stroke

The concept of stroke involves a heterogeneous group of processes. Ischemic stroke is caused by the obstruction of cerebral vessels and is the most common type of stroke, accounting for about 85% of the total. On the other hand, sudden bleeding in the brain induces a hemorrhagic stroke, accounting for the remaining cases [141]. Hemorrhagic stroke is due to bleeding into the brain by the rupture of a blood vessel, and it can be subdivided, depending on the localization of the blood vessels broken, into subarachnoid hemorrhage (SAH) and intracerebral hemorrhage (ICH). In SAH, the bleeding is into the subarachnoid space, while in ICH, the bleeding is into the brain parenchyma. Hemorrhagic stroke is associated with severe morbidity and high mortality, and it is related to worse outcomes, such as deterioration of consciousness and neurological dysfunction.

Ischemic stroke results in a sudden loss of oxygen and glucose to the brain tissue, leading to neuronal cell death and severe brain damage. It involves multiple processes, such as energy failure, alteration of the ion homeostasis, increased intracellular calcium levels, excitotoxicity, free-radicals toxicity, arachidonic acid generation, cytokine cytotoxicity, the BBB disruption, infiltration of leukocytes, inflammation, glial cells activation, and apoptosis, among others [142]. Meanwhile, the lack of energy supply leads to mitochondrial dysfunction and oxidative and nitrosative stress. Postischemic inflammation brings further damage to brain cells and tissues during reperfusion. These events aggravate the initial injury and ultimately lead to the death of endothelial cells, pericytes, glial cells, and neurons composing the NVU [143,144].

Oxidative stress significantly contributes to tissue injury in acute ischemic stroke. During ischemia, damaged electron transportation generates excessive superoxide (O_2_^-^), which facilitates the generation of other free radicals, such as H_2_O_2_ and hydroxyl radical (OH·). These reactive free radicals (ROS) further inhibit the mitochondrial electron transport, leading to even more ROS production [145,146]. Reperfusion also induces the production of O_2_^-^, nitric oxide (NO), and peroxynitrite. These free radicals are not only able to directly damage lipids, proteins, and nucleic acids to induce cell death but also activate MMPs to degrade collagen and laminins, which leads to vascular wall disruption and increased BBB permeability [142]. By activating the synthesis of transcription factors (e.g., NF-κB, hypoxia-inducible factor 1 (HIF-1), interferon regulatory factor 1 (IRF1), and signal transducer and activator of transcription 3 (STAT3)), oxidative stress is also able to induce numerous proinflammatory genes’ expression (e.g., ICAM-1, VCAM-1, E-selectin, and *P*-selectin). As cerebral levels of antioxidant enzymes and substances are not high enough, oxidative stress is relatively more harmful to the brain than other organs [143].

Inflammation further exacerbates stroke-induced tissue injury. Different types of cells, extracellular receptors, and inflammatory mediators participate in the inflammatory response after stroke. Inflammatory cells, such as microglia and astrocytes, participate in post-ischemic tissue remodeling. Increasing evidence shows that cerebral ischemia can activate microglia and astrocytes, which can release proinflammatory cytokines and chemokines, such as TNF-α, IL-1β, interleukin-6 (IL-6), and other cytotoxic molecules (e.g., NO and ROS) [147,148,149]. Moreover, ischemic stroke can cause the penetration of neutrophils and monocytes from the blood to the brain. Neutrophils are the earliest upregulated leukocyte subtype in the ischemic cerebral parenchyma [144], which triggers tissue damage by releasing ROS, proteases, and cytokines. Lymphocytes also participate in the inflammatory response after stroke and contribute to ischemia-induced damage [150].

CAMs facilitate leukocyte infiltration into the brain. During the inflammation process of an ischemic stroke, three classes of CAMs are activated: selectins, integrins, and immunoglobulins. During the early stage of ischemia, enhanced selectins, such as P-selectin and E-selectin, mediate leukocyte rolling and recruitment during inflammation [151]. Similarly, within hours after the onset of stroke, immunoglobulins, such as ICAM-1, can also be stimulated by cytokines secreted by microglia and astrocytes, among others [152]. In this context, soluble ICAM-1 (sICAM-1) has been proposed as an indicator for the severity of the stroke, as it is increased in acute ischemic stroke patients, and its expression level is significantly higher in patients who died than those who survived [153].

### 2.3. Alzheimer’s Disease and Cerebral Amyloid Angiopathy

AD is the most common cause of dementia worldwide. Aging is its principal risk factor, together with others, including smoking, obesity, head trauma, previous depression, female gender, positive family history, and several other conditions involving vascular-associated pathologies as diabetes mellitus, hypercholesterolemia, hypertension, atherosclerosis, coronary heart disease, and stroke [154,155]. AD can be originated by familial genetic alterations or sporadically, constituting a heterogeneous disorder. Several mutations have been described in the familial AD, accounting for those in the amyloid precursor protein (APP) and in presenilins 1 and 2 1 (PSEN1 and PSEN2) for most cases of the disease of this type [156,157]. However, the inheritance of the apolipoprotein E (apoE) ε4 allele is the main genetic risk factor in sporadic AD [158,159], together with aging and other environmental factors.

AD is a progressive and neurodegenerative disease. Neuropathologically, the intracellular accumulation of hyperphosphorylated tau protein as neurofibrillary tangles and neuropil threads, together with the extracellular accumulation of amyloid-β (Aβ) in the core of the neuritic plaques, are considered the two molecular and morphological signatures of AD. AD is also associated with microvascular dysfunction, neurovascular disintegration, defective BBB function and other vascular factors [124], which may contribute to the disease progression [160].

Although still under debate, different evidence supports the idea that Aβ deposition is the central event in AD pathogenesis, which latterly induces the formation of neurofibrillary tangles, cell injury, vascular damage, and ultimately dementia [161]. In the amyloidogenic pathway, β- and γ-secretases sequentially cleavage APP to generate Aβ peptides [162,163,164,165]. According to the amyloid hypothesis on the cause of AD, the initiating event of the pathological process is the imbalance between the clearance and production of Aβ. Subsequently, Aβ peptides over-accumulated at the extracellular level lead to neuronal degeneration and dementia [166].

In this regard, there have been described several molecular pathways responsible for the Aβ clearance, being the most relevant the proteolytic degradation by extracellular proteases, such as insulin-degrading enzyme (IDE), neprilysin (NEP), and endothelin-converting enzyme (ECE) [167], and through the perivascular clearance, which comprises perivascular drainage and glymphatic pathways [168]. Another mechanism for the Aβ clearing from the brain relies on the balance between the efflux and influx of Aβ across the BBB [169]. In this regard, two receptors are responsible for the Aβ transportation: the low-density lipoprotein receptor protein-1 (LRP-1) transports Aβ from the brain to blood [170], while the receptor for advanced glycation end products (RAGE) [171] does it from blood to the brain.

Besides the two main pathological hallmarks, extraneuronal Aβ plaques and neurofibrillary tangles, AD presents other traits, such as cerebral amyloid angiopathy (CAA) and inflammation. CAA is related to the accumulation of Aβ in the walls of arteries, arterioles, capillaries, and veins of the leptomeningeal and cortical regions [172,173]. In CAA, Aβ accumulates in cerebral blood vessels replacing smooth muscle cells and inducing vascular degeneration compromising the functionality and integrity of vessels and contributing to the BBB disruption [125].

In fact, CAA and AD pathology frequently co-occur, presumably because of the accumulation of Aβ in the brain [174]. However, independently from AD, the risk of symptomatic lobar ICH and microscopic cortical infarcts is the most relevant clinical consequence of CAA [175]. CAA patients also present cognitive impairment, lower perceptual speed and episodic memory impairment, separately from the effect of AD [176]. CAA is a sporadic disease associated with age, although familial CAA cases are reported as a consequence of mutations in the APP gene located within or just outside the Aβ coding region [174]. One of these mutations, for instance, causes the autosomal dominant disorder of Dutch-type hereditary CAA (also known as hereditary cerebral hemorrhage with amyloidosis (HCHWA)–Dutch type), which is clinically characterized by early-onset recurrent hemorrhagic strokes and dementia [177]. Nevertheless, the crosstalk between CAA and AD seems a clear example of the interactive effects of neurodegenerative and cerebrovascular diseases on cognition, which are likely a consequence of brain injuries caused by each process [174].

Wide bibliography emphasizes that vascular defects contribute to the onset and progression of neuronal degeneration and death in AD [178]. In this regard, the two-hit vascular hypothesis of AD incorporates a pathogenic vascular component to the excessive Aβ accumulation as initial events in the AD onset [132]. According to this hypothesis, vascular damage would impair the Aβ clearance, which would accumulate in cerebral vessels and parenchyma to generate toxicity [124,179]. This vascular damage would be induced mainly but not exclusively by cerebral hypoperfusion, NVU dysfunction or BBB disruption, for example, by CAA. Studies reinforcing this hypothesis have demonstrated that vascular abnormalities occur before changes in Aβ deposition, metabolic dysregulation, and functional impairment [180]. Hypoperfusion is detected at preclinical AD stages [181], and animal models of bilateral common carotid artery occlusion recapitulate AD pathology, including Aβ accumulation [182]. Several studies have observed deficiencies in the neurovascular response to various stimuli in MCI or early-stage AD, evidencing an NVU dysfunction, as recapitulated by Solis et al. [160]. In addition, increased BBB permeability is found in MCI and early AD patients, correlating with increased BBB leakage [128,183].

At the molecular level, mitochondrial function declines with aging, in parallel to enhanced production of intracellular oxidative agents, including ROS, and the expression of nitric oxide synthase (NOS). The ROS and the reactive nitrogen species jointly contribute to the malfunction of the BBB and injury to the cerebral parenchymal cells [184].

In addition, inflammatory factors are elevated in microvessels in AD [185], and Aβ is also able to induce the inflammatory cascade in human endothelial cells [186]. Cerebral endothelial activation inducing the expression of interleukins (IL-1β, IL-6, IL-8), vascular endothelial growth factor (VEGF), TNF-α, and MMPs, among others, occurs in AD altering brain homeostasis [139,187]. Elevated endothelial markers, such as E-selectin or VCAM-1, have been detected in the plasma of older subjects affected by late-onset AD and vascular dementia [139,188]. Moreover, activated microglia contributes to neuroinflammation and is associated with senile plaques in AD [189]. The presence of Aβ peptide in senile plaques of AD patients can also stimulate the secretion of proinflammatory cytokines and the complement system [190]. However, it also has been described that brain myeloid cells contribute to the Aβ removal through phagocytosis and that stimulation of the immune response in the CNS ameliorates Aβ deposition [191]. Taken together all these results, it can be concluded that AD can be correlated with factors, such as inflammation, besides Aβ deposition and neurofibrillary tangle formation [192].

## 3. SSAO/VAP-1 and Cerebrovascular Dysfunction

### 3.1. SSAO/VAP-1 in Stroke

To date, increasing evidence implicates that SSAO/VAP-1 may play an important role in stroke. In humans, several studies demonstrated that the plasmatic SSAO/VAP-1 is altered in the stroke condition, as listed in Table 4. In ischemic stroke, it has been reported that serum SSAO/VAP-1 protein increases in the acute phase (<6 h), while levels of the membrane-bound protein in the ipsilateral hemisphere decrease [193]. Other authors found a small increase in SSAO activity in plasma 24 h after ischemia but more significant and from 1 h after symptoms onset when patients developed a hemorrhagic transformation (HT) [92]. Interestingly, the levels of SSAO activity in plasma predict the adverse neurological outcome in ischemic stroke patients and also represent a robust predictor of the appearance of parenchymal hemorrhages after the treatment with tissue plasminogen activator (tPA) in these patients [92]. In this study, an increased SSAO activity was observed in the ipsilateral hemisphere, contrary to Airas et al. 2008 [193]. Differences at this level may be due to different ways to measure the protein, such as immunohistochemical approaches or enzymatic activity determination, or by the pathological condition itself. In this regard, the presence of HT may lead to massive infiltration of plasma content into the parenchyma, including SSAO/VAP-1, which may account for this increased activity in the ipsilateral hemisphere, as found by Hernandez-Guillamon et al. 2010, but may not be present in ischemic conditions without HT. Other authors had found no differences in plasma SSAO activity after ischemic stroke when samples were obtained more than 24 h after the onset of symptoms [93]. In this regard, the time of plasma obtention after the onset of symptoms may be crucial to detect differences, as plasma SSAO activity has been reported to decrease after the acute phase [92] or weeks after stroke [94]. Even so, in acute ICH, the increased plasma SSAO activity predicts neurological outcome, suggesting a possible contribution of the soluble protein in the secondary brain damage after the initial bleeding. Remarkably, patients clinically diagnosed as possible or probable CAA cases presented higher plasma SSAO/VAP-1 activity than patients who showed a hypertensive-related ICH [95]. On the other hand, serum SSAO activity levels are also associated with the appearance of cerebral microbleeds in MS [194].

Interestingly, the SSAO enzymatic inhibition in several embolic stroke models performed in rats diminishes the CAMs expression, downregulating the inflammatory reaction, the leukocyte adhesion and extravasation, decreasing the infarct volume, and recovering the neurological outcome [67,68,92,195,196], even in delayed cerebral ischemia [67,68,92,195,196]. Analogous anti-inflammatory activity and mitigated damage are found in animal models with suppressed SSAO activity or deficient in the SSAO/VAP-1 protein subjected to ischemia-reperfusion treatment in the lung [195] and heart [196], and the SSAO/VAP-1 inhibition is proposed as a novel therapy in ischemic acute kidney injury [65].

SSAO activity is able to upregulate the expression of other CAMs, such as E- and P-selectins, ICAM, or VCAM [63,197]. In addition, a possible role of soluble SSAO/VAP-1 has been suggested to spread the inflammatory signal from the ipsilateral side of the ischemic brain to the contralateral side [198]. These facts may reinforce the benefits of SSAO/VAP-1 inhibition on inflammation control under a stroke condition.

Moreover, using an in vitro experimental model of oxygen–glucose deprivation (OGD), Sun et al. described the role of SSAO/VAP-1 present in endothelial cells during ischemic stroke, which was consistent with results obtained in animal models. Different OGD and reoxygenation conditions were analyzed, and SSAO/VAP-1 presence increased the susceptibility of endothelial cells to the OGD insult. Under these conditions, the oxidation of its substrate through its enzymatic activity boosted the resulting damage on vascular cells, with the activation of caspases 3 and 8 during the cell death process. OGD also constituted a spur for the release of soluble SSAO/VAP-1, which was found to be mediated in part by MMP-2-dependent shedding. On the other hand, short times of OGD stimulated SSAO/VAP-1-dependent leukocyte binding on endothelium, a function that partially depends on its enzymatic activity [199].

Besides the beneficial effects of blocking leukocyte adhesion in stroke conditions, the inhibition of SSAO/VAP-1 activity also prevents the generation of H_2_O_2_, aldehydes and ammonia from its enzymatic catalyzation, which could contribute to oxidative stress in acute ischemic stroke when overproduced. In this regard, it has been reported that both the soluble [200] and the membrane-bound SSAO/VAP-1 could induce apoptosis in vascular cells through its oxidative metabolites [86], and this is enhanced under OGD conditions [199]. As brain cells are more sensitive to oxidative stress given their lack of antioxidants, good maintenance of the BBB tightness is crucial for preserving neuronal function. In this regard, the SSAO/VAP-1 inhibition could be beneficial in preserving the BBB by reducing the uncontrolled inflammation and maintaining the function of NVU after stroke.

### 3.2. SSAO/VAP-1 in Alzheimer’s Disease

As previously reported, SSAO/VAP-1 is expressed in vascularized tissues, including the brain [41,45,46,47,201]. The presence of SSAO/VAP-1 enzyme has also been determined in the brains of AD patients, as summarized in Table 4, and SSAO/VAP-1 immunoreactivity appeared restricted to meningeal and parenchymal blood vessels in the brain and markedly and selectively increased SSAO/VAP-1 immunoreactivity was observed to associate with vascular Aβ deposits in patients with AD. Moreover, augmented SSAO immunoreactivity appeared associated with elevated Cu/Zn superoxide dismutase 1 expression in abnormal blood vessels of diseased brains [96]. In parallel, circulating SSAO/VAP-1 was also assessed in the plasma of patients with sporadic AD. A clear rise in plasma SSAO activity was found in AD patients at moderate-severe and severe stages of the disease, compared to healthy controls [97]. No alteration of the enzyme was observed in AD patients with mild or moderate dementia compared with controls. Other authors have corroborated the plasma SSAO/VAP-1 increase in AD as well as in post-stroke dementia patients, negatively correlating with mini-mental state examination (MMSE) scores [98]. The elevation in plasmatic SSAO activity could be a consequence of its shedding from membrane-bound SSAO/VAP-1, particularly when the enzyme is overexpressed in AD [96]. Remarkably, plasma SSAO/VAP-1 levels did not correlate with Aβ in plasma samples [97]. Altogether, these results suggested that an elevated plasmatic SSAO activity could contribute to oxidative stress and vascular damage in advanced AD.

On the other hand, numerous studies have exposed that many physiopathological alterations are shared features between AD and diabetes mellitus (DM), including elevated cholesterol levels, aging-related processes, metabolic disorders, glycogen synthase kinase-3 elevated activity, aggregation of Aβ, association with cardiovascular diseases, increased oxidative stress, and inflammation response among others [99,100,101,102]. SSAO activity in the blood plasma of diabetic patients is also elevated [103]. Moreover, classic pathological signatures observed in AD are more prominent in diabetic patients, and DM constitutes a risk factor for AD [202,203]. In this context, the role of SSAO/VAP-1 was also assessed in human hippocampal vessels of non-demented DM, AD, and AD with diabetes mellitus (ADD) patients [204]. Results revealed enhanced accumulation of both SSAO/VAP-1 and Aβ immunolabeling intensity in vessels from ADD compared with AD patients. Interestingly, injured vessels exhibiting elevated SSAO/VAP-1 staining also presented augmented oxidative damage indicators and glial activation. Globally, this study suggests that increased vascular SSAO/VAP-1 levels in the human hippocampus may contribute to the faster pathology evolution in patients with both diseases.

Besides DM, other cardiovascular and lifestyle-related risk factors are increasingly accepted to be relevant for the pathogenesis of AD [205]. In this context, plasma SSAO/VAP-1 is positively associated with coronary artery disease, and its expression is increased in atherosclerotic plaques in humans and in ApoE-deficient mice [206]. Moreover, SSAO inhibition reduces atheroma, decreases oxidative stress in Apo-E-deficient mice, and attenuates the expression of adhesion molecules, chemoattractant proteins, and proinflammatory cytokines in the aorta. Thus, together with DM, the SSAO/VAP-1 alteration in several AD risk factors suggests that it could be not only involved in AD progression but also in the AD onset.

The aldehydes generated from methylamine metabolism by SSAO/VAP-1 are involved in protein unfolding and generate protein cross-linkages into lysine residues [207,208]. Therefore, it is reasonable to hypothesize that these compounds may react with lysines of Aβ to promote vascular aggregation of Aβ in AD patients. In this regard, the potential effects of these SSAO-generated endogenous aldehydes have been implicated in Aβ misfolding, oligomerization, and fibrillogenesis [209,210]. In fact, formaldehyde concentrations are elevated in senescence-accelerated mouse-prone 8 (SAMP8) mice, which correlates with cognitive dysfunction, and is associated with increased SSAO activity of these mice [211]. In addition, urine formaldehyde measurement has been proposed as a biomarker for AD and post-stroke dementia progression as, in parallel to plasma SSAO/VAP-1 content, it negatively correlates with MMSE scores [98]. These results suggest that increased SSAO/VAP-1 expression, as well as its circulating form, maybe a source of oxidative stress in the blood vessel wall in AD. Moreover, considering that SSAO/VAP-1 is overexpressed in cerebrovascular tissue of patients with CAA-AD, and its intrinsic enzymatic activity generates pro-aggregating metabolites, one can conclude that SSAO/VAP-1 may contribute to the vascular damage associated with AD [212,213].

**Table 4:** Alterations of SSAO/VAP-1 levels found in human ischemic stroke, intracerebral hemorrhage (ICH) and Alzheimer’s disease (AD).

Disorder	Tissue Analyzed	Phase of the Pathology	SSAO/VAP-1 Alteration	Reference
Ischemic stroke	Serum	<6 h (acute phase)	Increase	[193]
	Plasma	24 h after stroke	Increase vs. 1 h	[92]
	Plasma	1 h after HT	Increase	[92]
	Serum	>24 h after stroke	No change	[93]
	Plasma	weeks after	Decrease	[94]
	Ipsilateral brain	-	Decrease	[193]
	Ipsilateral brain	-	Increase	[92]
Hemorrhagic stroke (ICH)	Plasma	3–4 h after ICH	Increase	[95]
	Contralateral brain	-	Increase	[95]
AD	Plasma	moderate-severe	Increase	[97]
	Plasma	-	Increase	[98]
	Brain vessels	-	Increase	[96]
	Hippocampus	-	Increase	[98]
	Brain vessels	-	Increase	[204]
	Brain vessels	-	Increase	[212]

**Figure 1:**
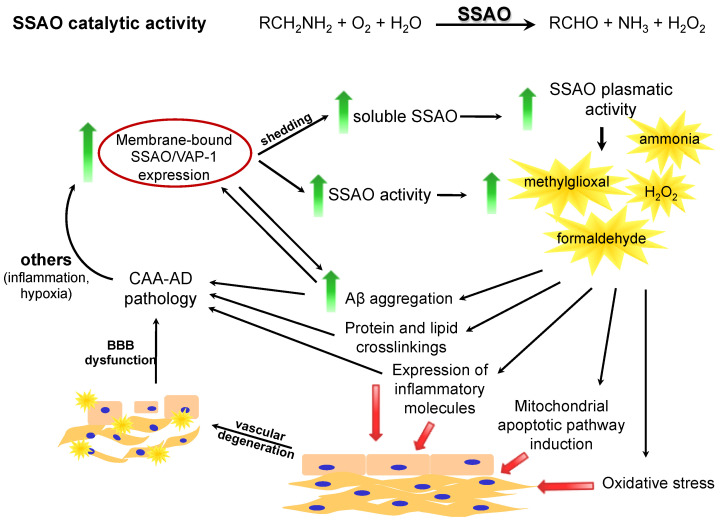
Summary of the pathogenic mechanisms of SSAO/VAP-1 in exacerbating the progress of cerebral amyloid angiopathy (CAA)-AD. Through the generation of toxic metabolites from SSAO activity (ammonia, methylgioxal, formaldehyde, and H_2_O_2_), SSAO/VAP-1 induces the vascular degeneration on both endothelial and smooth muscle cells through several mechanisms: (i) oxidative stress, (ii) induction of apoptosis through the mitochondrial pathway, (iii) induction of the expression of pro-inflammatory molecules (selectins, VCAM, ICAM…), (iv) induction of protein and lipid crosslinking, and (v) increase in Aβ aggregation. The resulting vascular degeneration, together with the protein and lipid crosslinkage and the Aβ aggregation contribute to the vascular degeneration and the CAA pathology, and these generate a positive feedback loop reinforcing SSAO/VAP-1 overexpression. Aβ aggregation itself also contributes to the SSAO/VAP-1 increase.

As shown in Figure 1, the abilities of SSAO metabolic products (e.g., H_2_O_2_, ammonia, and aldehydes) to generate oxidative stress, to enhance the AGEs generation, to promote the Aβ aggregation, and to induce apoptosis, reinforce the role of SSAO/VAP-1 in CAA-AD-related vascular pathology.

On the other hand, other functions attributed to endothelial SSAO/VAP-1 also may play a role in promoting or aggravating the pathology of AD, including those related to granulocytes binding and leukocyte trafficking into tissues. In this regard, different peripheral inflammatory cells have been detected in brains from AD and animal models: monocytes, lymphocytes and neutrophils [214]. The adhesion function of SSAO/VAP-1 is particularly active for neutrophils. Neutrophils infiltrate the brain parenchyma in AD and migrate towards Aβ deposits in the experimental mouse model [215]. However, this neutrophil infiltration resulted in an exacerbation of microgliosis and behavioral deficits in an experimental AD model [216]. More recently, it has been demonstrated that neutrophil adhesion in brain capillaries may impair cognitive functions [217]. These and other studies performed in mouse AD models indicate that neutrophils may contribute to the initial stages of the disease [214,218]. Although the specific participation of SSAO/VAP-1 has not been assessed in these models, it is reasonable to believe that the potential inhibition of leukocyte trafficking may be beneficial in AD. Thus, the regulation of VAP-1 may also be considered as a strategy to address this aspect associated with the pathology.

From this background, elucidating whether vascular SSAO/VAP-1 modulation is a consequence, or a cause of specific pathologic processes seems a question that needs to be solved. However, the study of this enzyme is difficult since the primary culture of SSAO/VAP-1-positive cells gradually loses its expression, and immortalized cell lines do not display activity or expression of SSAO [34,48]. In this concern, newly developed vascular cell lines stably expressing the human SSAO/VAP-1 have been established [219,220,221]. The transfected protein is essentially expressed as a dimer in the vascular cells membrane, and specifically, localize in the lipid rafts of these cells. The protein levels and enzymatic activity, and kinetic parameters of the enzyme in these cells are similar to those detected in vivo by the same cell types. These new SSAO/VAP-1-expressing endothelial cell lines (HUVEC, human umbilical vein endothelial cells, and hCMEC/D3, human cerebral microvascular endothelial cells) are also able to mediate leukocyte adhesion, a known function of SSAO/VAP-1 in endothelium under inflammatory conditions that are not observed in smooth muscle cells [222]. Thus, these new cell lines constitute suitable experimental tools for studying new functions of SSAO/VAP-1, as well as to elucidate its role in pathological processes or to evaluate potential molecules that could modify its activity for therapeutic purposes [222].

To reveal the nature of the SSAO/VAP-1 relationship with the AD pathology, cell lines stably expressing human SSAO/VAP-1 were treated with different Aβ forms to simulate the CAA conditions in vitro [220]. The vasculotropic Dutch-mutated Aβ1-40 (Aβ_1-40_D) peptide, which accumulates in vessels of the Dutch-type hereditary CAA brain, was used to reproduce the pathology in vitro. It was found that the treatment with Aβ_1-40_D increased the vascular SSAO/VAP-1-dependent toxicity, which was correlated by a rise of SSAO/VAP-1 protein in the membrane of endothelial cells. Moreover, SSAO/VAP-1 enhanced the deposition of Aβ on vascular cells by both activity-dependent and -independent mechanisms. Taken together, these data suggest that Aβ itself can be one of the elements stimulating the SSAO/VAP-1 elevation in AD, augmenting its toxic action, and inducing the vascular dysfunction and, in turn, that SSAO/VAP-1 can stimulate Aβ deposition on the vascular walls, thereby contributing to the CAA-AD progression.

As previously mentioned, the function and structure of both NVU and BBB are abundantly compromised in several neurological diseases, such as stroke and [223]. In this context, it is necessary to study the neurovascular crosstalk and alterations to better comprehend the molecular base of AD [123]. Therefore, a new in vitro model of NVU was used to assess the possible contribution of vascular SSAO/VAP-1 overexpression to the BBB dysfunction through its role on endothelial activation, the modification of angioneurins release and the alteration of the NVU communication [224]. As human umbilical venous endothelial cells, HUVECs are different from those present in the endothelium of the BBB. To better mimic the NVU model, the cell line hCMEC/D3 [225] was stably transfected with hSSAO/VAP-1 [221]. With this model, it was interesting to decipher the role of SSAO/VAP-1 in endothelial activation, the angioneurins release, the BBB permeability, the BBB function alteration, and the Aβ deposition. Using the hSSAO/VAP-1-expressing hCMEC/D3 cells, co-cultured with mixed mouse neuron-glia primary cultures as an experimental model of NVU, it was observed that SSAO/VAP-1 induced the endothelial activation by modifying the release of proinflammatory and pro-angiogenic angioneurins IL-6, IL-8, and VEGF. In parallel, the alteration of the BBB structure was also exhibited a decreased level of tight-junction proteins, such as zona ocludens-1 and claudin-5. The activation of signaling pathways by the products of SSAO catalytic activity or the structural modifications induced by only the presence of this enzyme could be the molecular mechanisms responsible for regulating these phenotypic changes. An increasing permeability and leukocyte adhesion, as well as an augmentation of Aβ deposition, were also observed by both enzymatic activity-dependent and independent mechanisms [224]. These results revealed that the expression of SSAO/VAP-1 in human brain microvessels induces an endothelial activation status towards a proinflammatory phenotype, accompanied by BBB leakage and leukocyte adhesion. In addition, the proinflammatory molecules released by the enzymatic activity-independent mechanisms could affect the surrounding microenvironment, the neighboring cells, and thus the NVU. Furthermore, the SSAO activity in enhancing leukocyte adhesion and Aβ deposition on endothelial cells suggests SSAO/VAP-1 inhibition, a promising strategy to bring beneficial effects for the treatment of AD [224].

## 4. Can SSAO/VAP-1 Be a Link between Stroke and AD?

### 4.1. Stroke and the Risk for AD

Mounting evidence suggests that disorders affecting the vascular system, as can be cerebrovascular diseases, including stroke, play a significant part in the development and progress of neurological diseases like AD in the elderly [123,139,226,227]. The existence of a strong link between vascular damage and AD is evidenced by a high percentage of patients who have suffered strokes and subsequently develop AD [228,229]. In this regard, several reports revealed that brain stroke/ischemia significantly rises the occurrence of AD [226,230,231]. This risk is even more elevated in cases where other vascular risk factors, for example, atherosclerosis, coexist with stroke [226]. Several mechanisms may contribute to this fate, as recently reviewed by Goulay et al. [227].

Both hypoxia and ischemic injury induce upregulation of Aβ generation, confirming the link between stroke and AD [228,229]. Evidence also indicates that increased APP accumulation is present at areas of ischemic brain damage in models of middle cerebral artery occlusion (MCAO) or focal cerebral ischemia. Thus, the APP cleavage may be induced under ischemic conditions [230,231,232,233,234,235,236,237]. Moreover, hypoxia induces the increase of BACE-1 expression and activity to increase the Aβ generation. Prolonged hypoxia can also induce mitochondrial dysfunction, neuronal loss, and potential memory deficits, facilitating the pathogenesis of AD [238,239]. Mechanistic studies reveal that both oxidative stress and HIF-1 are accountable for the elevated expression of beta-secretase 1 (BACE-1) at distinct phases after cerebral ischemia [240].

Besides increasing Aβ generation, ischemic conditions can alter the Aβ clearance by inducing downregulation of the levels of NEP and ECE-1 in the brain, enzymes that are responsible for the degradation of Aβ [241,242]. In addition, several mechanisms can harm the BBB after hypoxia/ischemia insults, including altering the expression of major Aβ clearance enzyme LRP-1 to impair the clearance of Aβ from the brain [243]. Moreover, the expression of RAGE, another important protein in the BBB that transports Aβ across BBB to the brain, is upregulated in the brains of mice undergone an experimental stroke or systematic hypoxia, thus diminishing the Aβ clearance from the brain [244]. On the other hand, it has been described that transient hypoxia damage can increase the hyperphosphorylation of tau in cortical neurons [245,246].

Ischemic or hemorrhagic stroke frequently involves the breakdown of the BBB, and as a consequence, soluble plasmatic proteins reach brain parenchyma, triggering inflammation and subsequent neurodegeneration [247,248]. In addition, the excessive release of free radicals and oxidative environment generated as a result of stroke injury are well-known contributors to the development and progression of AD [249,250].

Among the common features between stroke and AD, restricted brain perfusion, cerebrovasculature dysfunction, and inflammation are the most significant ones, which finally lead to neuronal injury and cognitive impairment [251]. All these data allow us to conclude that it exists a robust association between AD and cerebrovascular disease, and the injury effects of stroke, through numerous pathways, could facilitate neurodegeneration, worsen dementia, and the outcome of AD.

### 4.2. AD and the Risk for Stroke

Cerebral hypoperfusion, atherosclerosis, oxidative stress, and vascular Aβ deposition around the cerebral vascular wall as CAA are alterations found in AD that can lead to an acute cerebrovascular functional failure by way of brain ischemia or hemorrhage [240]. CAA directly compromises cerebrovascular function, causing not only cerebral hypoperfusion, and therefore, chronic ischemia but also BBB disruption and micro-bleeds [252,253]. In fact, CAA is associated with hemorrhagic stroke [254], and symptomatic patients with CAA often present with lobar intraparenchymal hemorrhages [255].

At the molecular level, Aβ presence decreases the expression of tight junction proteins in experimental models and in human samples, which is associated with BBB leakage [256,257]. Under these conditions, MMP-2 and MMP-9 are upregulated and degrade the cerebral basement membrane and lead to cerebral hemorrhage [258].

Inflammation acts as another important contributor to neurovascular dysfunction since the main source of vascular reactive oxygen species contributing to the Aβ-associated cerebral blood flow disturbances are perivascular macrophages reacting to vascular Aβ [259]. Vascular Aβ deposits promote the migration of monocytes across BBB [260], which generate oxidative stress and proinflammatory cytokines [261]. Clusters of activated microglia are also present around vascular Aβ deposits [262]. These cells create an inflammatory response to release proinflammatory cytokines and oxidative stress mediators that induce the loss of BBB integrity by the disruption of tight junctions [263]. In addition, Aβ deposition on vascular walls activates cell signaling pathways in endothelial and smooth muscle vascular cells that contribute to the BBB disruption, as apoptotic cell death pathways, generation of free radicals, and disruption of intracellular Ca^2+^ homeostasis [264]. These vascular cells are also able to release proinflammatory cytokines that are upregulated in AD, contribute to the CAA progression and the subsequent development of hemorrhages. The specific mechanism of this vascular cells-mediated inflammatory response is not clearly defined, but it is thought to contribute to the BBB breakdown [265].

### 4.3. SSAO/VAP-1 As a Possible Link between Stroke and AD

SSAO/VAP-1, which is present in the cerebrovasculature, and particularly in the endothelial and smooth muscle cells, is a common factor involved in the pathogenesis of stroke and AD, but may also be a possible link between both diseases, mediating the onset of one of them in patients affected by the other.

On one hand, evidence has exposed that SSAO/VAP-1 is abnormally elevated in the cerebrovascular tissue, colocalizing with Aβ deposits in AD patients [96,206,214]. There, SSAO/VAP-1 is able to promote aggregation of Aβ, vascular cell injury through its enzymatic activity, and lead to the BBB dysfunction [211,215,222,228]. In addition, in smooth muscle cells from brain meninges, excessive SSAO/VAP-1 activity would contribute to the Aβ aggregation and extracellular matrix cross-linkage, inducing the rigidity of the vessel and the final breakdown generating hemorrhage. The increased SSAO/VAP-1 present in blood plasma has already been associated with vessel wall breaking, for instance, in ischemic stroke patients undergoing HT [92]. Given these considerations, it is reasonable to deduce that under AD conditions, the alterations induced by SSAO/VAP-1 could easily drive to a vascular wall weakening and, therefore, to an increased risk of suffering a subsequent hemorrhagic stroke in the presence of AD.

On the other hand, SSAO/VAP-1 participates in the inflammatory response in multiple models of stroke [67,92,195], mediating leukocyte-endothelium adhesion and upregulating other CAMs [63,199,266,267], augmenting the leukocyte adhesion cascade, and producing toxic and pro-aggregating products, such as H_2_O_2_ and formaldehyde [62]. These products are directly involved in the oxidative stress and Aβ aggregation responsible for AD pathology. Thus, the SSAO/VAP-1 alterations produced during a stroke may contribute to generating the conditions necessary for the AD onset in stroke patients: a high pro-oxidant, pro-aggregating and inflammatory environment. The increased plasma SSAO/VAP-1 presence described in post-stroke dementia patients [98] reinforces this hypothesis. Although more research should be done to establish the SSAO/VAP-1 alterations as a causative link between stroke and AD, the evidence existing so far suggests that SSAO/VAP-1 could participate in the transition from stroke to AD or from AD to stroke.

## 5. Therapeutic Approach to Stroke and AD by SSAO/VAP-1 Inhibition

The vast bibliography on the association of SSAO/VAP-1 with DM, atherosclerosis, AD, or stroke has suggested this enzyme as an important therapeutic target for these diseases [195]. Moreover, there is a wide consensus in describing AD pathogenesis as a multifaceted neurological disorder, which development and evolution may be prompted by several mechanisms embracing cholinergic dysfunction, oxidative stress and free radicals formation, disproportionate protein misfolding and aggregation, biometal dyshomeostasis, excitotoxicity, and neuroinflammation, in addition to disturbances in the monoaminergic and glutamatergic systems. Furthermore, the increase of BACE-1 induced by both hypoxia and ischemic damage enhances the Aβ generation, confirming the link between AD and stroke [228,240].

Because modifications of the levels of plasmatic SSAO/VAP-1 humans have been associated with several pathological conditions such as ischemic stroke and AD [92,95,96,193], it would be interesting to design new molecules able to inhibit SSAO/VAP-1 and to interact with other molecular systems involved in AD and stroke, as a novel therapeutic option.

Drugs currently approved by the US Food and Drug Administration (FDA) drugs for the treatment of AD-associated cognitive deficits are grounded on the cholinergic hypothesis of AD with limited therapeutic interest [268,269]. Given the limited effectivity of anticholinergic therapies as well as the multifactorial and extreme complexity of AD nature, several researchers have suggested a new idea, based on “one molecule, multiple targets,” also known as the multi-target directed-ligand (MTDL) approach, which proposes the beneficial use of molecules with numerous pharmacological profiles that would allow them to interact with diverse molecular targets [266,270,271,272,273].

**Figure 2:**
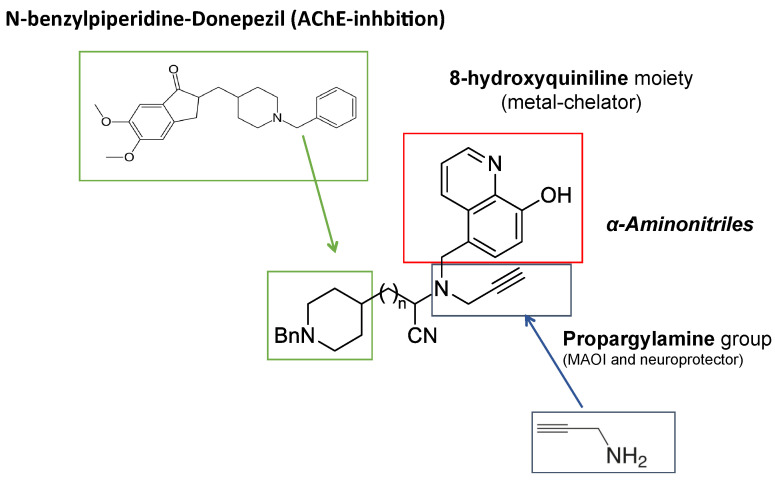
Structure of the multi-target directed-ligand (MTDL) donepezil-propargylamine-8-hydroxyquinoline, DPH4 [221].

Regarding the new series of MTDL molecules designed to be used in AD therapy, it was interesting to analyze their effect on cerebral ischemia as well. A novel series of molecules grown on the hybridization of selected moieties from donepezil, propargylamine, and 8-hydroxyquinoline (DPH), were synthesized and pharmacologically assessed for the prevention of AD [267]. Among them, the DPH4 (Figure 2) resulted in being a good MAO-A, MAO-B, acetylcholinesterase (AChE), and butyrylcholinesterase (BuChE) inhibitor, and besides, DPH4 displayed robust biometal chelating properties against Cu^2+^ and Fe^2+^, good absorption, distribution, metabolism, excretion, and toxicity (ADMET) properties confirming its interesting properties to be used in AD therapy. In this context, the possible protective effect of DPH4 in cerebral ischemia was studied, as well.

Using an experimental model of ischemic stroke, the cell viability of the human cerebral microvascular endothelial cells stably expressing the hSSAO/VAP-1 was assessed after OGD and reoxygenation in the presence of DPH4. Under these hypoxic conditions, a release of soluble SSAO/VAP-1 was observed, contributing to the vascular cell damage through its catalytic action. DPH4 pretreatment mediated a dose-dependent protective effect on hSSAO/VAP-1-expressing hCMEC/D3 cells in the presence of methylamine in both normoxic and OGD with reoxygenation conditions. The beneficial action of DPH4 was significant on inflammation also, as DPH4 significantly diminished the adhesion of leukocytes to the endothelium in the presence of methylamine, as a consequence of its inhibitory action on SSAO activity. To simulate a pre-existing AD pathology, Aβ_1-40_D treatment was introduced into this experimental model of ischemia. DPH4 showed a protective effect against the synergistic damaging effect induced by methylamine and Aβ_1-40_D. These results not only confirmed the important role that SSAO/VAP-1 plays in enhancing endothelial cell death under ischemia but also suggest that DPH4, a new MTDL molecule containing donepezil, propargylamine, and 8-hydroxyquinoline, is able to protect brain endothelial cells under hypoxia through its inhibitory and anti-inflammatory activity on SSAO/VAP-1, and may be used for AD therapy [221].

Moreover, a new series of molecules, such as indole substituted hydrazides and hydrazines, were synthesized as potential MAO inhibitors in vitro [274,275], and have been analyzed of their multipotent inhibitory potency towards MAOs A and B, SSAO/VAP-1, AChE and BuChE. Among them, the hydrazine JL72 (3-(3-hydrazinylpropyl)-1H-indole) exhibited a potent, reversible inhibitory activity on MAO-A, which suggests its ability to restore serotoninergic neurotransmission. Moreover, it behaved as a moderate BuChE inhibitor and as a high-affinity inhibitor towards SSAO/VAP-1. The molecule JL-72 also showed significant anti-inflammatory activity in HUVEC hSSAO/VAP-1-expressing cells by measuring leukocyte adhesion on the endothelium. Taken together these results, JL-72 resulted in being a good candidate lead compound for the subsequent development of drugs targeting cerebrovascular and neurological diseases, such as stroke and AD [21].

The neuroprotective effect of statins has been widely reported in the therapy of neurodegenerative diseases such as Parkinson’s disease, AD, and vascular dementia [276]. Regarding their beneficial effects observed in stroke, these effects have been described to be independent of their role in cholesterol reduction [277]. Two different animal models of stroke (embolic MCAO through the injection of a clot, eMCAO; and transient MCAO by introducing an intraluminal filament, tMCAO [278], and an in vitro model of stroke using human brain microvascular endothelial cells expressing SSAO/VAP-1 under OGD, were used to assess the possible involvement of SSAO/VAP-1 in the protective effect of simvastatin. In the animal model, the soluble SSAO/VAP-1 is released into the bloodstream after an ischemic stimulus, correlating with an increase in E-selectin, VCAM-1, and with the infarct volume. Simvastatin blocked soluble SSAO/VAP-1 release and prevented E-selectin and VCAM-1 overexpression, and also effectively blocked SSAO/VAP-1-mediated leukocyte adhesion. The attenuation of SSAO/VAP-1 release could be responsible for the beneficial effects of simvastatin observed in protecting against the proinflammatory effects of ischemia in these animal models. Similar results were also observed in cultured cell lines and, therefore, highlight the importance of the attenuation of SSAO/VAP-1-dependent inflammatory response in the process [198].

## 6. Conclusions

All these data allow us to conclude that a robust connection between cerebrovascular diseases and AD exists, and the adverse effects of the stroke condition, through multiple pathways, could enable the progression to neurodegeneration and the outcome of AD. On the other hand, AD could also facilitate the appearance of stroke and aggravate the outcome following strokes. Most interestingly, based on the data discussed above, SSAO/VAP-1 could be a common link between both pathologies. In this context, the design of new MTDL molecules being able to inhibit SSAO/VAP-1 activity and interact with the cholinergic and monoaminergic system could be a promising therapeutic approach for the treatment of both stroke and AD.

## Figures and Tables

**Table 2:** SSAO/VAP-1 is involved in the binding/transmigration of different types of leukocytes, but not of other types. This selectivity also depends on the organ studied and the inflammatory stimulus.

Inflammatory Stimulus	Organ/Tissue	Type of Leukocytes Bound by SSAO/VAP-1	Type of Leukocytes not Bound by SSAO/VAP-1	Reference
Ischemia/reperfusion	Kidney	Neutrophils	Macrophages/T-lymphocytes	[65]
Postischemic inflammation	Brain	Neutrophils	-	[66]
Subarachnoid hemorrhage	Brain	Neutrophils	-	[67]
Intracerebral hemorrhage	Brain	Neutrophils	-	[68]
Peritonitis	Peritoneum	Granulocytes	-	
Air pouch inflammation	Subcutaneous	Monocytes/lymphocytes	-	[69]
LPS	Brain	Neutrophils	-	[70]
LPS, *Klebsiella* pneumoniae	Lungs	Polymorphonuclear cells, neutrophils	-	[71]
Acute liver failure	Liver	Leukocytes	Monocytes	[72]
ConA hepatitis	Liver	CD4+ Th2 cells	-	[73]
Hepatic chronic inflammation and fibrosis	Liver	CD16+ monocytes	-	[74]
Liver inflammation	Liver	CD4+ T cell	-	[75]
Liver allograft rejection	Liver	CD4+ and CD8+ lymphocytes	-	[76,77]
Tumors (adhesion function)	Skin	CD45+, CD3+, CD8+	CD4+, T-reg cells, Type2 macrophages, GR-1+CD11b+	[78]
Tumors (enzymatic function)	Skin	CD45+, CD8+, CD11b+, granulocytes,	CD4+, type2 macrophages	[78]
Cytokine-induced angiogenesis	Eyes	CD11b+ cells, granulocytes	-	[79]
Diabetic retinopathy	Eyes	Leukocytes	-	[80]
Uveitis	Eyes	CD45+	-	[81]
In vitro	Endothelial cells	Lymphocytes, T-killer cells	Neutrophils, monocytes	[82]
In vitro	Endothelial cells	Polymorphonuclear leukocytes	-	[83]
AOC3 knockout	Adipose tissue	CD45+, T cells, macrophages, natural killer	-	[84]

**Table 3:** Physiological functions of SSAO/VAP-1 and pathological effects associated with these functions in situations where the enzyme is overexpressed. Data are summarized from [7,8,9,16,17,18,31,32,33,34,78,87,88,89,90,91,92,93,94,95,96,97,98,99,100,101,102,103,104].

Physiological Function	Pathological Effect Upon SSAO/VAP-1 Overexpression	Involvement in Pathologies
Oxidative deamination of primary amines of endogenous and xenobiotic origin Molecular signaling through H_2_O_2_ generation	Toxicity of metabolic products (formaldehyde, methylglyoxal, H_2_O_2_)	Stroke AD
	Protein cross-linking and Aβ aggregation	Diabetes
	Oxidative stress	Atherosclerosis
	AGEs generation	Congestive heart failure
	Inflammation	Fibrotic liver disease
	Pathological angiogenesis	Cancer Age-related macular degeneration
Leukocyte trafficking under inflammatory conditions	Excessive inflammatory response	MS
Insulinomimetic action by recruitment of GLUT4 receptors to the cell membrane	Unknown	Unknown

## Data Availability

Not applicable.

## Abbreviations

Aβ	Amyloid-β
AChE	Acetylcholinesterase
AD	Alzheimer’s disease
ADD	AD with diabetes mellitus
ADMET	Absorption, distribution, metabolism, excretion, and toxicity
AGEs	Advanced glycation end products
AOs	Amine oxidases
apoE	Apolipoprotein E
APP	Amyloid precursor protein
BACE-1	Beta-secretase 1
BBB	Blood–brain barrier
BuChE	Butyrylcholinesterase
CAA	Cerebral amyloid angiopathy
CAMs	Cell adhesion molecules
CNS	Central nervous system
DAO	Diamine oxidase
DM	Diabetes mellitus
DMBA	7,12-dimethylbenz(alpha)anthracene
EAE	Experimental autoimmune encephalomyelitis
ECE	Endothelin-converting enzyme
eMCAO	Embolic MCAO
FAD	Flavin adenine dinucleotide
FDA	Food and Drug Administration
GLUT	Glucose transporters
H_2_O_2_	Hydrogen peroxide
HCHWA	Hereditary cerebral hemorrhage with amyloidosis
hCMEC/D3	Human cerebral microvascular endothelial cells
HEVs	High endothelial venules
HIF-1	Hypoxia-inducible factor 1
HT	Hemorrhagic transformation
HUVEC	Human umbilical vein endothelial cells
ICAM-1	Intracellular adhesion molecule 1
ICH	Intracerebral hemorrhage
IDE	Insulin-degrading enzyme
IL-1β	Interleukin 1 beta
IL-6	Interleukin 6
IFN- γ	Interferon- γ
IRF1	Interferon regulatory factor 1
JL-72	3-(3-hydrazinylpropyl)-1H-indole
JNK	c-Jun amino-terminal kinase
LOX	Lysyl oxidase
LPS	Lipopolysaccharide
LRP-1	Lipoprotein receptor protein-1
LTQ	Lysine tyrosyl quinone
MAO	Monoamine oxidase
MAPK	Mitogen-activated protein kinase
MCAO	Middle cerebral artery occlusion
MCI	Mild cognitive impairment
MMPs	Matrix metalloproteinases
MMSE	Mini-mental state examination
MS	Multiple sclerosis
MTDL	Multi-target directed-ligand
NEP	Neprilysin
NO	Nitric oxide
NOS	Nitric oxide synthase
NVU	Neurovascular unit
O_2_^-^	Superoxide
OGD	Oxygen–glucose deprivation
OH·	Hydroxyl radical
PAO	Polyamine oxidase
PLN	Peripheral lymph node
PNAd	Peripheral lymph addressin
PrAO	Primary amine oxidase
PSEN	Presenilin
RAGE	Receptor for advanced glycation end products
ROS	Reactive free radicals
SAH	Subarachnoid hemorrhage
SAMP8	Senescence accelerated mouse-prone 8
SSAO	Semicarbazide-sensitive amine oxidase
STAT3	Signal transducer and activator of transcription 3
tMCAO	Transient MCAO
TNF-α	Tumor necrosis factor α
tPA	Tissue plasminogen activator
TPQ	Topa-quinone
VAP-1	Vascular adhesion protein-1
VCAM-1	Vascular cell adhesion protein 1
VEGF	Vascular endothelial growth factor
